# Supplementary material for: Effects of Seed Bio-Priming by Purple Non-Sulfur Bacteria (PNSB) on the Root Development of Rice
Source: Microorganisms. 2022 Nov 6;10(11):2197. doi: 10.3390/microorganisms10112197 (PMC9698004; doi:10.3390/microorganisms10112197)
Supplement: Supplementary file 1 [file microorganisms-10-02197-s001.zip › Figure S1 Seed bio-priming revised.pptx]

## Slide 1
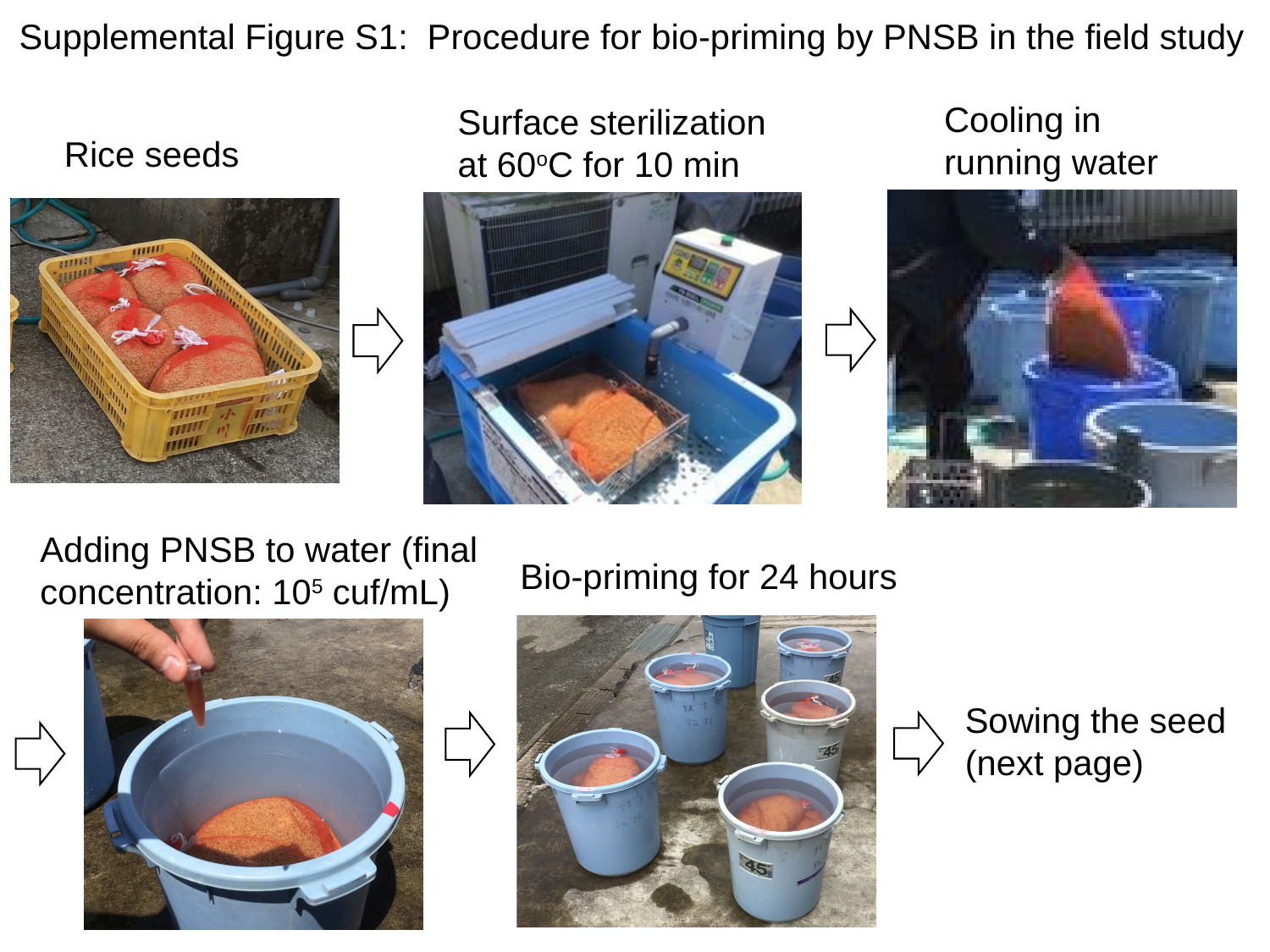

Supplemental Figure S1: Procedure for bio-priming by PNSB in the field study
Cooling in running water
Surface sterilization at 60oC for 10 min
Rice seeds
Adding PNSB to water (final concentration: 105 cuf/mL)
Bio-priming for 24 hours
Sowing the seed
(next page)

## Slide 2
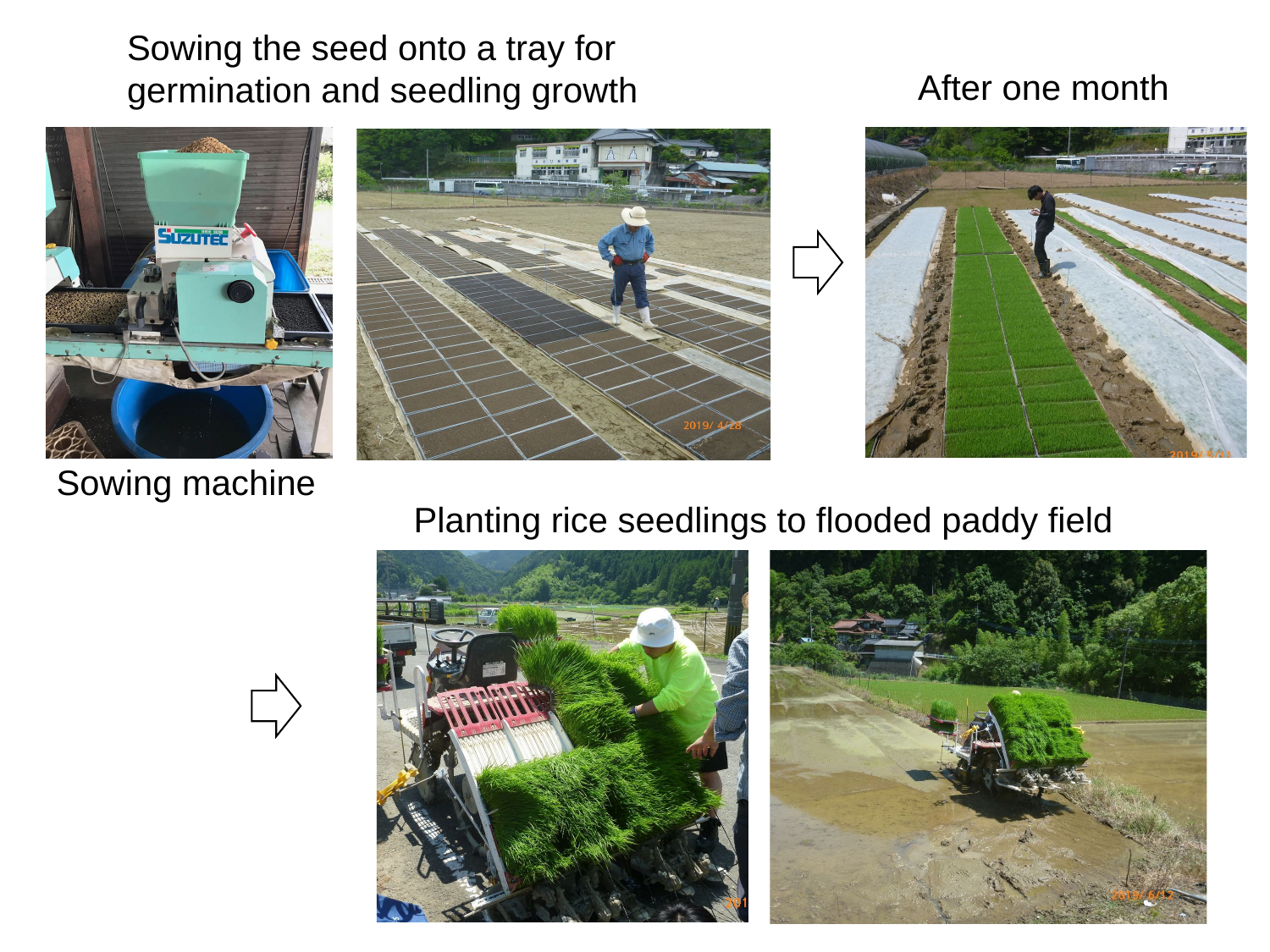

Sowing the seed onto a tray for germination and seedling growth
After one month
Sowing machine
Planting rice seedlings to flooded paddy field
