# Supplementary material for: Effects of Seed Bio-Priming by Purple Non-Sulfur Bacteria (PNSB) on the Root Development of Rice
Source: Microorganisms. 2022 Nov 6;10(11):2197. doi: 10.3390/microorganisms10112197 (PMC9698004; doi:10.3390/microorganisms10112197)
Supplement: Supplementary file 1 [file microorganisms-10-02197-s001.zip › Figure S2 revied for upload.pptx]

## Slide 1
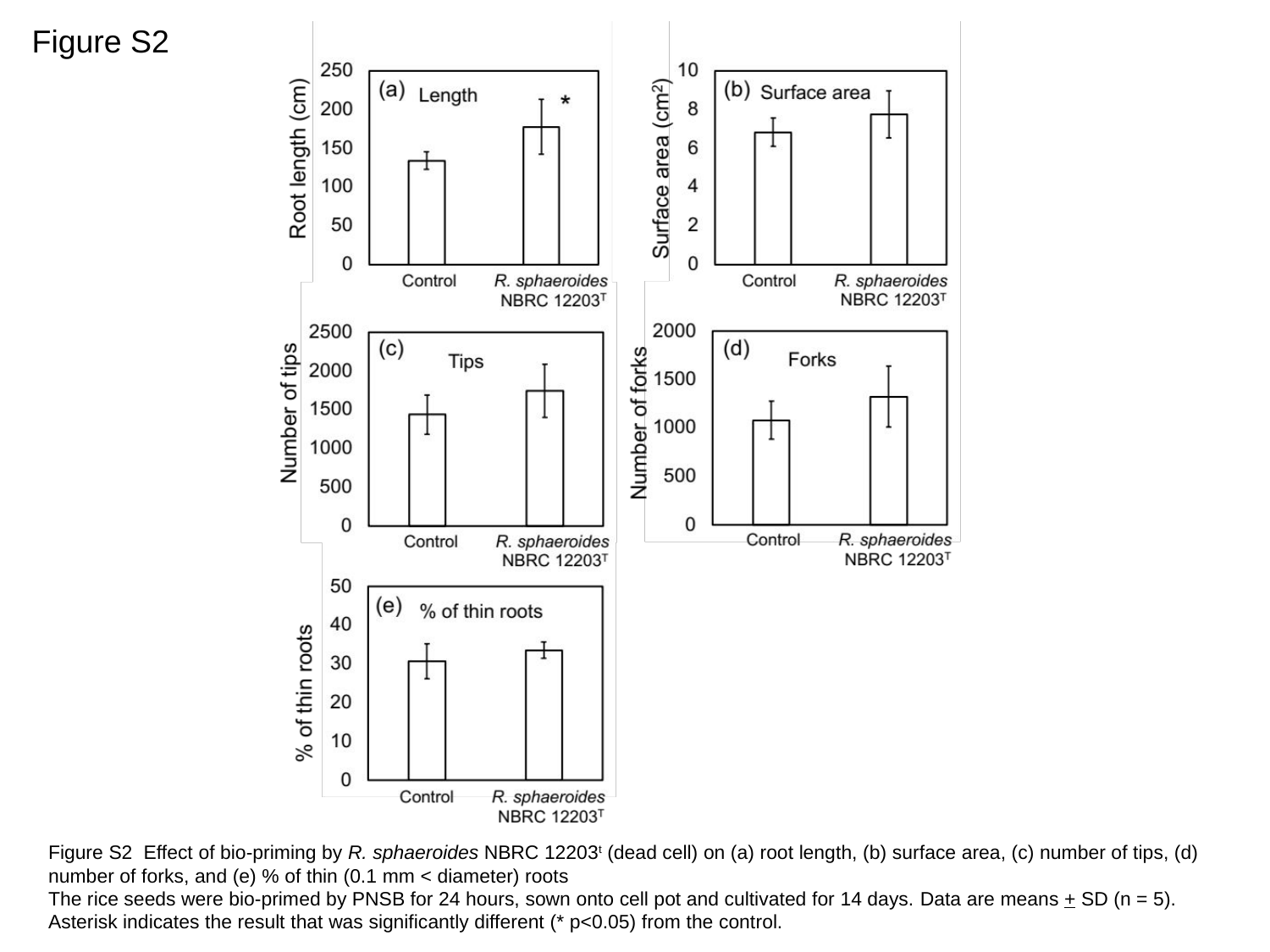

Figure S2
Figure S2 Effect of bio-priming by R. sphaeroides NBRC 12203t (dead cell) on (a) root length, (b) surface area, (c) number of tips, (d) number of forks, and (e) % of thin (0.1 mm < diameter) roots
The rice seeds were bio-primed by PNSB for 24 hours, sown onto cell pot and cultivated for 14 days. Data are means + SD (n = 5). Asterisk indicates the result that was significantly different (* p<0.05) from the control.
